# Analysis of PD-1 expression in the monocyte subsets from non-septic and septic preterm neonates

**DOI:** 10.1371/journal.pone.0186819

**Published:** 2017-10-19

**Authors:** Magdalena Zasada, Marzena Lenart, Magdalena Rutkowska-Zapała, Małgorzata Stec, Wojciech Durlak, Andrzej Grudzień, Agnieszka Krzeczkowska, Nina Mól, Marta Pilch, Maciej Siedlar, Przemko Kwinta

**Affiliations:** 1 Department of Paediatrics, Institute of Paediatrics, Faculty of Medicine, Jagiellonian University Medical College, Krakow, Poland; 2 Department of Clinical Immunology, Institute of Paediatrics, Faculty of Medicine, Jagiellonian University Medical College, Krakow, Poland; Children's Hospital of Los Angeles, UNITED STATES

## Abstract

Programmed death-1 (PD-1) receptor system represents a part of recently reported immunoregulatory pathway. PD-1 is an immune checkpoint molecule, which plays an important role in downregulating the immune system proinflammatory activity. Until recently, PD-1 expression was not established on immune cells of the preterm infants. The study objectives were to confirm expression of the PD-1 receptors on the monocytes isolated from very low birth weight newborns (VLBW), and to analyze their expression during the first week of life and late-onset sepsis. Peripheral blood mononuclear cells were isolated from 76 VLBW patients without early-onset sepsis on their 5^th^ day of life (DOL). PD-1 expression was determined on the monocyte subsets (classical, intermediate, non-classical) by flow cytometry. In case of late-onset sepsis (LOS), the same analysis was performed. Our results demonstrated that on the 5^th^ DOL, PD-1 receptors were present in all the monocyte subsets. Children, whose mothers had received antenatal steroids, presented higher absolute numbers of non-classical monocytes with PD-1 expression. Infants born extremely preterm who later developed LOS, initially showed a lower percentage of PD-1 receptor-positive intermediate monocytes in comparison to neonates born very preterm. During LOS, we observed a rise in the percentage of classical monocytes with PD-1 expression. In case of septic shock or fatal outcome, there was a higher percentage and absolute count of intermediate monocytes with PD-1 expression in comparison to children without these complications. In conclusion, monocytes from VLBW children express PD-1 receptors. Antenatal steroid administration seems to induce PD-1 receptor expression in the non-classical monocytes. PD-1 might play a role in immunosuppressive phase of sepsis in the prematurely born children with septic shock and fatal outcome.

## Introduction

Neonates, especially those born preterm, are highly susceptible to systemic infections [1]. Developmental functional immaturity of the immune system is regarded as one of the reasons for the high rate of sepsis in the prematurely born patients [2]. Circulating monocytes (MO) are important elements of the innate immunity. MO in the peripheral blood can be divided into three subsets according to the CD14 and CD16 antigen surface expression: CD14^++^CD16^-^ (classical, CL MO), CD14^++^CD16^+^ (intermediate, IM MO) and CD14^+^CD16^++^ (non-classical, NC MO) [3]. The major population are CL MO, which constitute about 90% of the entire monocyte pool, whereas the IM and NC MO subsets account for about 10% under physiological conditions in adults [4]. CL MO show high phagocytic activity. The CD16^+^ MO population (IM and NC) can significantly increase during infection and inflammation [5], which was documented both in adult [6], as well as neonatal [7] patients with bacterial sepsis.

High rates of morbidity and mortality in sepsis result from a prolonged immunosuppression after a rapid proinflammatory response; it is associated with attenuated pathogen clearance and/or susceptibility to superinfection [8]. The programmed death receptor-1 (PD-1) pathway seems to play a role in sepsis-induced immune suppression [9]. PD-1 (CD279) is a cell surface receptor that belongs to the immunoglobulin superfamily, first described by Ishida and colleagues in 1992 [10]. It is one of the key negative regulators of the immune response, maintains immune tolerance [11], prevents development of autoimmune diseases [12], and controls extend of healthy tissue damage during infection [13]. It is an inducible molecule expressed on the surface of activated cells, mainly T cells, B cells, NK cells, but also monocytes and dendritic cells [14–15]. A negative signal transmitted from the activated PD-1 receptor leads to decreased activity of the immune system by inhibition of the TCR or BCR connected signaling pathways, decreased production of the cytokines and proteins promoting immune cell survival (e.g. Bcl-2), and increased synthesis of IL-10, which inhibits the immune response [13].

MO from adult subjects during sepsis display increased expression of PD-1 simultaneously with decreased HLA-DR expression. Inhibition of PD-1 in the pre-clinical studies appears to restore defects of the immune functions and to improve survival in sepsis [16–18].

To our knowledge, expression of the PD-1 receptors on the circulating monocytes has never been investigated in prematurely born infants. Here, we would like to present results of a prospective cohort study designed to determine expression changes of PD-1 receptors on the monocyte subpopulations in the very low birth weight (VLBW) preterm children soon after birth and in the subgroup with late-onset sepsis (LOS).

## Materials and methods

### Study design and setting

This was a prospective observational study performed at a 30-bed Neonatal Intensive Care Unit (NICU), Department of Paediatrics, Institute of Paediatrics, Faculty of Medicine, Jagiellonian University Medical College, Krakow, Poland, between 2014 and 2016. Protocol of the study was approved by the Jagiellonian University Ethic Committee.

### Patient selection

The newborns enrolled to this study were recruited from the patients consecutively admitted to NICU.

Inclusion criteria were:

1. Signed informed consent by the parents,
2. Birthweight < 1500g,
3. Gestational age (GA) ≤ 32 weeks,
4. Age ≤ 72 hours at the time of enrolment.

Exclusion criteria were:

1. Early-onset sepsis (defined as a blood culture-proven clinical sepsis occurring up to 3 days of life),
2. Multiple congenital malformations.

### Monitoring during hospitalization

All the subjects enrolled in the study underwent careful clinical monitoring for symptoms of LOS. LOS was defined as a blood culture-proven clinical sepsis occurring ≥ 72 hours of age. Clinical symptoms of LOS were first identified by an attending neonatologist, and then verified with a positive blood culture. Septic shock definition was based on this mentioned in the Surviving Sepsis Campaign, adjusted for age range based on the criteria of Goldstein as determined by the consensus [19–20].

### Data collection

Data regarding baby’s perinatal history, hospitalization course, laboratory results and other LOS pertaining data were collected in our hospital database.

### Blood collection/Division into groups

First sample of blood was drawn from all the study participants on the 5^th^ day of life (DOL). The blood sample (500 μl) was collected into EDTA-containing tubes (Vacutainer System^®^, Becton Dickinson Biosciences, San Jose) and processed in the laboratory at Department of Clinical Immunology, Institute of Pediatrics, Faculty of Medicine, Jagiellonian University Medical College, Krakow, within 2 hours from the draw. For this study, the samples were retrospectively categorized into 2 groups depending on whether the patients did or did not develop LOS during their hospitalization:

- Non-LOS group—samples collected on the 5^th^ DOL from newborns, who did not develop LOS during hospitalization in the NICU
- Before-LOS group—samples collected on the 5^th^ DOL from newborns, who developed the episode of culture-proven LOS during hospitalization in the NICU.

Children, who were diagnosed with LOS during hospitalization, had their second blood sample collected within 24 hours from the onset of LOS symptoms. Once LOS was confirmed by a positive blood culture, these samples formally formed a third, LOS-group.

### Flow cytometry

**Fig 1:**
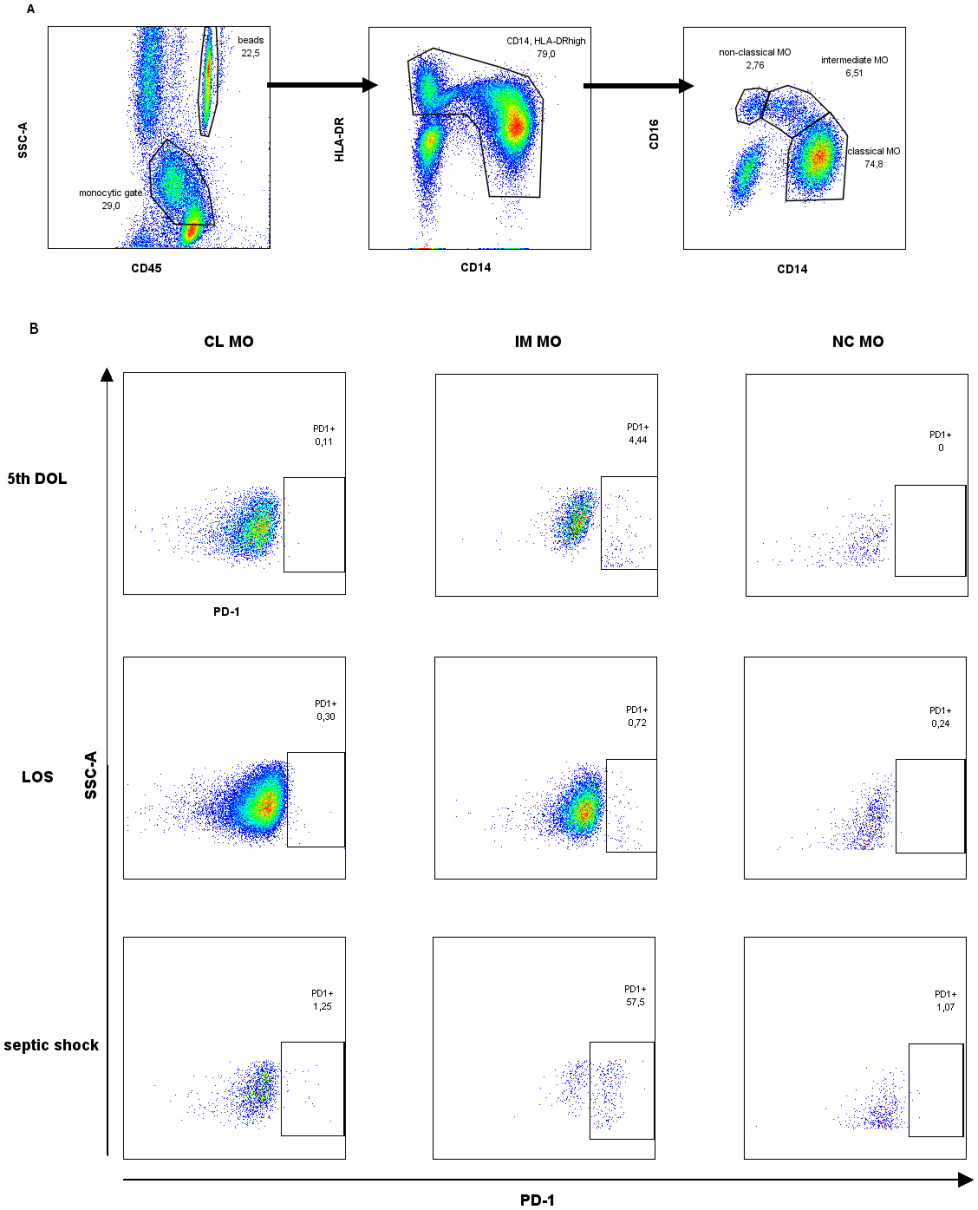
Gating strategy for studying monocyte subsets and differential expression of PD-1 in monocyte subsets. (A) Gating strategy of monocyte subsets. The CD45-positive monocytes (“monocytic gate”) together with adjacent lymphocytes were further analyzed. The cells were then gated to exclude CD14-HLA-DR- NK cells and finally divided into CD14++CD16- (classical), CD14++CD16+ (intermediate) and CD14+CD16++ (non-classical) monocytes. The absolute numbers of monocyte subsets were estimated based on acquired bead count (“beads gate” on plot no 1). (B) PD-1 expression on monocyte subsets. PD-1 expression pattern is shown on each of the monocyte subsets (classical, intermediate and non-classical subsets) in a patient analyzed on 5^th^ DOL, a patient that developed LOS, and a patient with septic shock.

Undiluted whole blood samples were washed by addition of 3 ml of 0.9% NaCl in polypropylene round-bottom tubes (BD Biosciences) and centrifuged (1000 × g). Then, blood sample was put into a TruCOUNT^™^ Tube (BD Biosciences, San Jose, USA) and stained on ice for 30 min with: anti-CD45-APC, anti- HLA-DR-PerCP, anti-CD14-FITC, anti-CD16-PE (BD Biosciences) and anti-PD1-PE-Cy7 (eBioscience) monoclonal antibodies (mAbs). The samples were then treated with FACS Lysing Solution (BD) until erythrocytes were lysed, and the cells were immediately processed in the FACSCanto flow cytometer (Becton Dickinson, San Jose, USA) along with 10.000 of beads per tube. The absolute numbers of CD14^++^CD16^-^, CD14^++^CD16^+^ and CD14^+^CD16^++^ monocytes were calculated with reference to the bead count. The percentage and the absolute numbers of PD-1- positive cells were estimated in each of the monocyte subset. Flow cytometric data were analyzed using FlowJo software (Tree Star, Inc, Ashland, OR). The gating strategy and analysis of MO subsets was previously described by us [21] and others [22] and is presented on Fig 1.

### Statistical analysis

Basic demographic data were compared using the Wilcoxon test or two-sided t-test as appropriate. Qualitative values were compared by the chi-square test. Student’s t-test was used to establish differences in the continuous variables with normal distribution between studied groups. For data with distribution other than normal, Wilcoxon test was used. Extreme values defined as lower than Q1-3IQR, or as higher than Q3+3IQR were excluded from the analyses. A probability value of p<0.05 was considered statistically significant. JMP^®^ 13.1.0 (SAS Institute Inc., 2016) was used for statistical analysis.

## Results

### Study population

A group of 76 preterm-born infants without early-onset sepsis with mean gestational age 27.7 (SD 2.4) weeks and mean birth weight 1042 (SD 257) grams was enrolled to the study. Thirty nine infants developed LOS during hospitalization, mostly caused by Gram-positive bacteria. LOS occurred on 17^th^ DOL (IQR 12–21.5 DOL; range: 8–34 DOL). Five infants diagnosed with LOS developed septic shock, and a total of eight patients from the entire study group died during hospitalization at NICU (Table 1).

**Table 1:** Comparison of selected demographic variables and hospitalization data of the patients in the two studied groups.

	Group non-LOS n = 37	Group before-LOS n = 39	P value and statistical test used
**Perinatal history**			
Birth weight [g], mean (SD)	1142 (241)	948 (237)	0.0011^T^
Gestational age [weeks], mean (SD)	28.6 (2.2)	26.8 (2.3)	0.0015^T^
Male gender	21 (57%)	16 (41%)	0.169^C^
Vaginal delivery	13 (35%)	10 (26%)	0.407^C^
Antenatal steroids	9 (24%)	12 (31%)	0.577^C^
**Hospitalization**			
Peripheral lymphocyte count on 5^th^ DOL [cells/μl], median (IQR)	2640.5 (1256.5–3363.8)	1964.5 (1420–2350)	0.350^W^
Late-onset sepsis	-	39	
Gram-positive	-	28 (72%)	
Gram-negative	-	7 (18%)	
Polymicrobial	-	4 (10%)	
Septic shock	-	5 (13%)	
**Outcome**			
Death during hospitalization at NICU	2 (5%)	6 (15%)	0.147^C^

^T^–two-sided T test,

C—Chi-square test,

^W^–Wilcoxon test.

DOL—day of life, NICU—Neonatal Intensive Care Unit

### Comparison of median levels of monocyte PD-1 expression on 5^th^ day of life

Analysis of the blood collected on the 5^th^ DOL showed that the majority of the isolated monocytes constituted the CL subtype, whereas IM and NC monocytes were present in smaller counts. There were cells positive for PD-1 receptors’ expression in all the monocyte subsets on the 5^th^ DOL. The IM MO subset showed the highest expression of PD-1 receptor (Table 2).

**Table 2:** Counts of monocyte subsets, monocyte subsets with PD-1 expression, and a percentage of PD-1^+^ cells within each monocyte subset in the blood samples from VLBW infants collected on the 5^th^ DOL. Data presented as median and IQR.

	CL MO	IM MO	NC MO	p
MO count (cell/μl); median [IQR]	850 [463–1921]	75 [35–134]	41 [12–106]	p < 0.0001*, Post-hoc tests**: p < 0.0001 for CL MO vs. IM MO, p < 0.0001 for CL MO vs. NC MO, p = 0.0009 for IM MO vs. NC MO
PD-1-positivecount (cell/μl); median[IQR]	2 [0.2–5.42]	8.5 [3–15]	0.33 [0–2]	p < 0.0001*, Post-hoc tests**: p < 0.0001 for CL MO vs. IM MO, p = 0.0011 for CL MO vs. NC MO, p < 0.0001 for IM MO vs. NC MO
PD-1-positive (%); median [IQR]	0.29 [0.05–0.58]	11.6 [6.34–22.6]	1.53 [0.17–4.81]	p < 0.0001*, Post-hoc tests**: p < 0.0001 for CL MO vs. IM MO, p < 0.0001 for CL MO vs. NC MO, p < 0.0001 for IM MO vs. NC MO

*—Kruskal—Wallis test,

**—nonparametric comparisons for each pair using Wilcoxon method

CL MO—classical monocytes, IM MO—intermediate monocytes, NC MO—non-classical monocytes

We did not find significant differences in percentages or numbers of the MO subsets expressing PD-1 receptor in regards to as function of the newborns’ gender or mode of their delivery. Interestingly, even though there was no difference in absolute counts of any MO subset in infants whose mothers had received prenatal steroids (n = 21) compared to those who had not undergo such a treatment, they had a significantly higher absolute count of NC MO expressing PD-1 (1000/ml [0–5000] vs. 110/ml [0–1000]; Wilcoxon, p = 0.0371).

**Fig 2:**
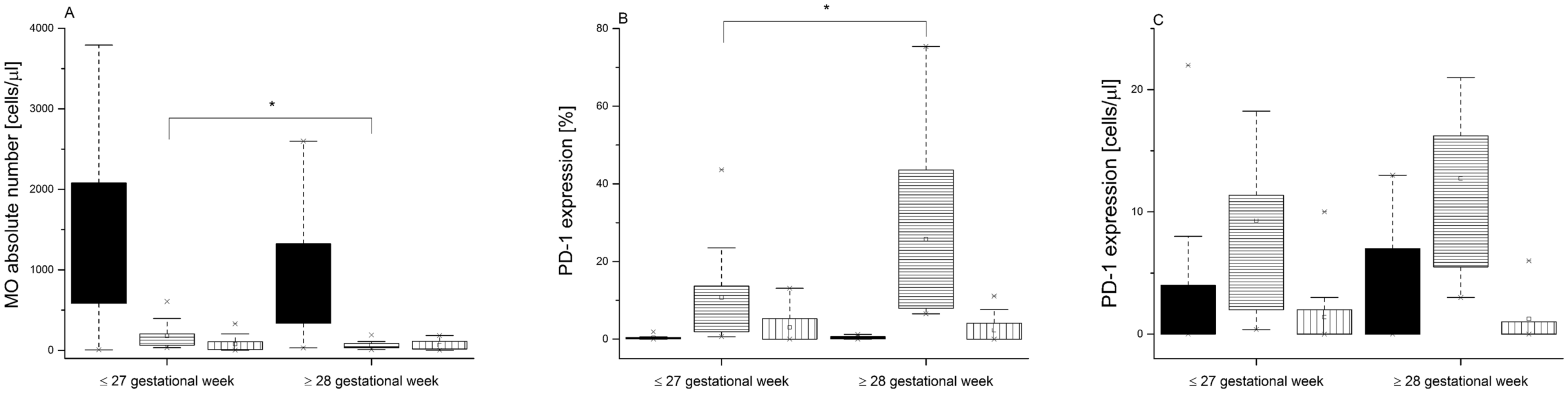
Monocyte populations and PD-1 expression in the before-LOS group of VLBW infants. Patients were subdivided into groups based on the level of their gestational age at birth. (A) Absolute numbers of monocyte subsets. (B) Percentages of PD-1 expressing monocytes. (C) Absolute numbers of PD-1 expressing monocytes. Data presented as median and IQR (box), compared with Wilcoxon test. Whiskers—range within 1.5 IQR. Classical monocytes are presented as a black graph, intermediate monocytes are presented as a graph with horizontal lines, whereas non-classical monocytes are presented as a graph with vertical lines. P-value was significant in case of *p<0.05.

Following LOS diagnosis, the study group was retrospectively divided into two groups with or without sepsis. There was no difference in PD-1 expression on the 5^th^ DOL in the non-LOS and before-LOS populations. There was also no correlation between PD-1 expression and corresponding gestational age on the 5^th^ DOL. However, we noticed that level of maturity at birth affected PD-1 expression in the before-LOS group. The neonates born as extremely preterm (≤ 27 gestational week) presented higher absolute counts of the IM MO (101/μl [58–288] vs. 59/μl [33–89]; Wilcoxon, p = 0.0105) and lower percentages of IM PD-1-positive MO (7.95% [1.8–14] vs. 15.8% [7.7–45.7]; Wilcoxon, p = 0.0136) in comparison to these who were born very preterm (≥ 28 gestational week) (Fig 2).

### PD-1 expression levels in patients with late-onset sepsis

**Fig 3:**
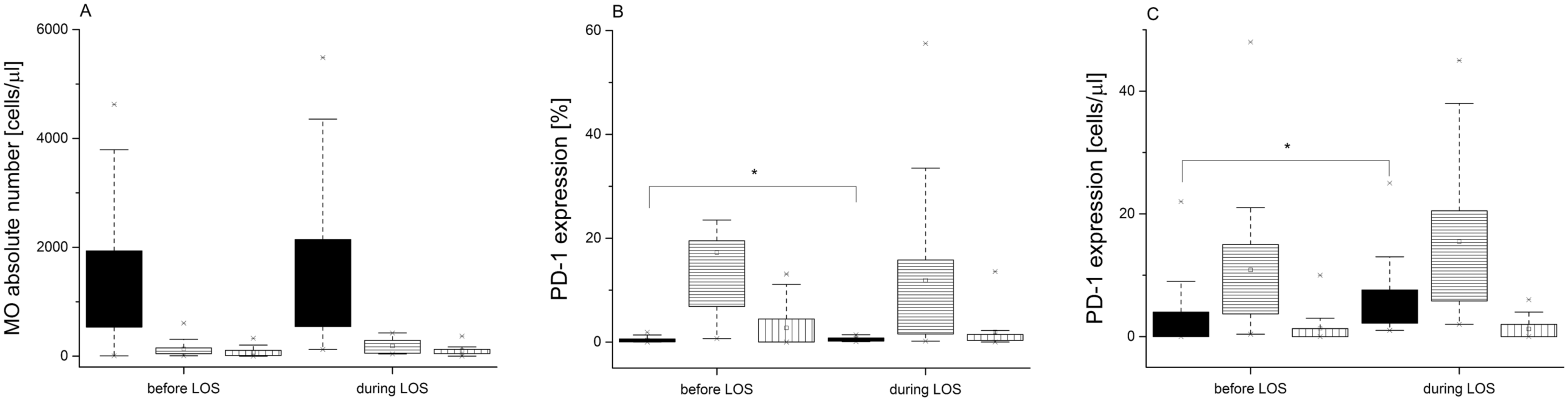
Monocyte subsets and PD-1 expression in the VLBW infants before and during late-onset sepsis. (A) Absolute numbers of monocyte subsets. (B) Percentages of PD-1 expressing monocytes. (C) Absolute counts of PD-1 positive cells Data presented as median and IQR (box), compared with Wilcoxon test. Whiskers—range within 1.5 IQR. Classical monocytes are presented as a black graph, intermediate monocytes are presented as a graph with horizontal lines, whereas non-classical monocytes are presented as a graph with vertical lines. P-value was significant in case of *p<0.05.

The next step in our study was to analyze the MO subsets and PD-1 expression in children, who developed LOS during hospitalization. We observed increase in absolute count of IM and NC MO during LOS in comparison with the results on the 5^th^ DOL, although the above differences did not reach statistical significance. Moreover, there was an increase in percentage (LOS group vs. before-LOS group: 0.47% [0.017–0.85] vs. 0.23% [0.04–0.61]; Wilcoxon, p = 0.043) and absolute count (LOS group vs. before-LOS group: 3.53/μl [2.1–8.3] vs. 2.24/μl [0–4.25]; Wilcoxon p = 0.0177) of the CL MO with PD-1 expression (Fig 3).

There was no difference in PD-1 expression on the circulating MO during LOS between samples taken from the children with either Gram-positive or Gram-negative systemic infections.

### PD-1 expression levels in LOS patients with septic shock and/or fatal outcome

**Fig 4:**
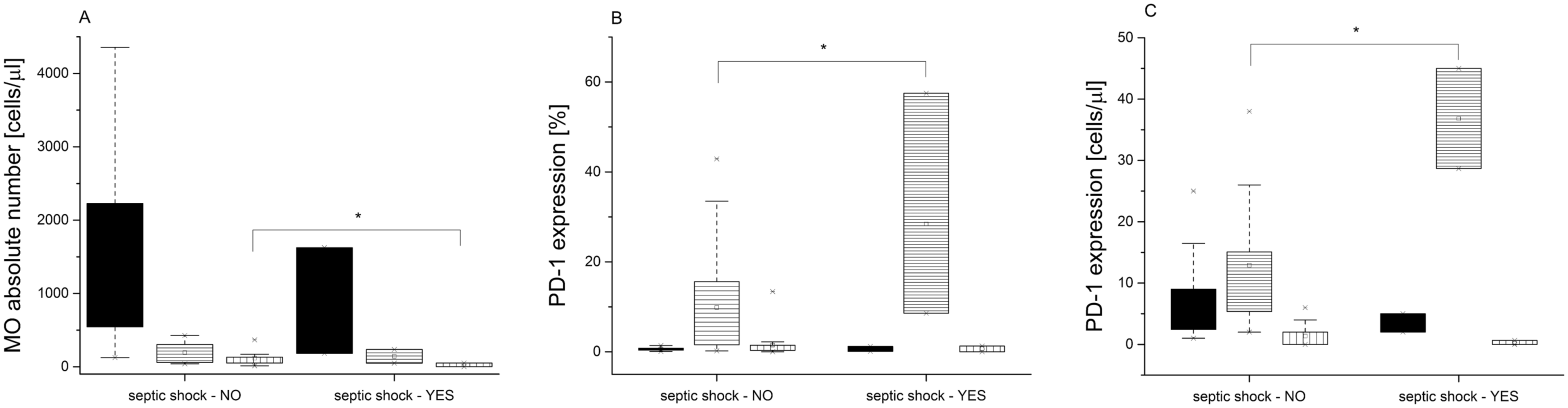
Analysis of the monocyte subpopulations in the patients with or without septic shock. (A) Absolute numbers of monocyte subsets. (B) Percentages of PD-1 positive cells. (C) Absolute numbers of PD-1 positive cells. Data presented as median and IQR (box), compared with Wilcoxon test. Whiskers—range within 1.5 IQR. Classical monocytes are presented as a black graph, intermediate monocytes are presented as a graph with horizontal lines, whereas non-classical monocytes are presented as a graph with vertical lines. P-value was significant in case of *p<0.05.

In a LOS-group, we observed significant differences in MO counts and PD-1 expression between patients who developed septic shock (n = 5) vs. those without this complication (n = 34). We observed a lower absolute number of NC subset in septic shock patients (31/μl [2–51] vs. 88/μl [51–134]; Wilcoxon, p = 0.0386). Moreover, they presented with a higher percentage (19.3% [8.59–57.5] vs. 4.44% [1.57–15.6]; Wilcoxon, p = 0.0498) as well as increased absolute number of the IM monocytes with PD-1 expression (36.8 cells/μl [28.7–45] vs. 12 cells/μl [5.2–15.5]; Wilcoxon, p = 0.0335) in comparison to these, whose sepsis was not complicated by septic shock (Fig 4).

**Fig 5:**
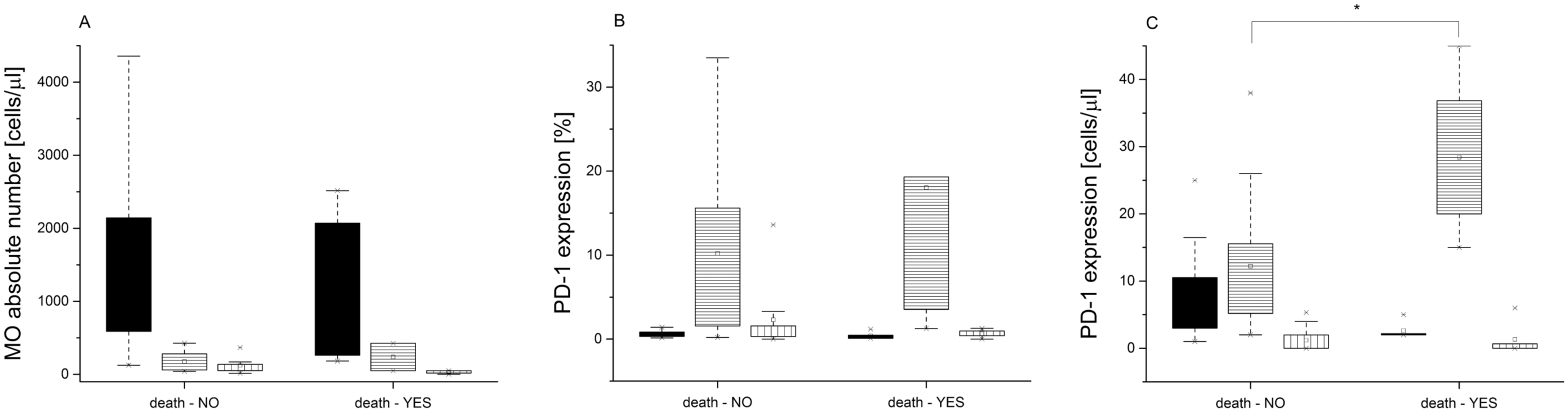
Analysis of the monocyte subpopulations in VLBW infants who either did nor did not survived LOS. (A) Absolute numbers of monocyte subsets. (B) Percentages of PD-1-positive cells. (C) Absolute numbers of PD-1 positive cells. Data presented as median and IQR (box), compared with Wilcoxon test. Whiskers—range within 1.5 IQR. Classical monocytes are presented as a black graph, intermediate monocytes are presented as a graph with horizontal lines, whereas non-classical monocytes are presented as a graph with vertical lines. P-value was significant in case of *p<0.05.

Finally, we demonstrated significant increase in the absolute number of PD-1 positive IM MO in the terminal patients compared to the infants who survived (Fig 5).

## Discussion

In our study, we showed that PD-1 receptor is expressed on the monocytes isolated from prematurely born VLBW children. We analyzed monocyte subsets in the above population on their 5^th^ DOL and during LOS.

The analysis of MO isolated from the VLBW infants five days after birth revealed that the CL subset constituted the majority of the monocytes, whereas IM and NC MO were present in smaller numbers. It was previously reported that the CL MO subset was predominant under stable clinical condition and without ongoing sepsis in adult population [23]. Our results matched also the findings of another study by Wisgrill et al. [24] who showed that the majority of the monocytes from the cord blood of the VLBW babies constituted the CL MO subset.

We would like to emphasize our novel finding which demonstrates that variable PD-1 expression is dependent on the maturation state of the monocytes and that PD-1 receptor is present on the monocytes—cells that should present the ligand.

PD-1 expression is a marker of cell exhaustion [25], but PD-1 holds several other important functions, such as regulation of the immune tolerance phenomenon and normal processes of the immune cells such as their differentiation/maturation and activation [26]. Therefore, PD-1 expression on the immune cells’ surface appeared to be justified from the biological point of view even in the premature infants. Monocytes are a source of tissue macrophages that drive the inflammatory process [27]. It is hypothesized that a PD-1 dependent mechanism can limit the pro-inflammatory activity of these cells within tissues. Furthermore, the PD-1 dependent mechanism may also contribute to the production and release of pro-inflammatory cytokines in microcirculation by CD16^+^ monocytes.

In our study, infants with a lower gestational age (≤27 gestational weeks) presented higher absolute number of IM MO and had lower percentage of PD-1 positive IM MO in comparison to the cells isolated from the children born ≥28 gestational week. A potential link between lower gestational age and higher count of IM MO was also observed by the previously cited study by Wisgrill et al [24]. However, the reasons of the observed shift towards the IM MO subset in the preterm neonates require further studies. It was suggested that IM MO chemokine receptor pattern indicated their possible role in angiogenesis [28]. On the other hand, since they typically increased in inflammatory state, their higher number might also indicate inflammation that initially caused premature labor.

Taking into consideration our finding that PD-1 expression was diminished on the IM MO in the study population, we suggest the following explanation. According to the exome analysis of the sorted MO subsets, the IM and NC MO display a higher apoptotic potential than the CL MO subset [29], [30]. In concordance with the hypothesis of Zhao at al. [29] this finding may highlight a mechanism used to limit the pro-inflammatory response associated with CD16^+^ monocytes. As the PD-1 pathway is important in triggering apoptosis [31], our study supports and expands the above mentioned theory. We further propose that the apoptosis of CD16+ MOs is dependent on the level of PD-1 expression. As such, before differentiation into the NC MO, the IM MOs with high PD-1 expression may have already undergone apoptosis, and therein cannot be observed within the NC MO pool. Such a response may be beneficial in neonates who present with a constitutionally lower fraction of PD-1-positive IM MO. These neonates are more likely to have an early proinflammatory response, even to subliminal infectious agents, as the mechanism of elimination of major proinflammatory cytokine manufacturers is less effective. In this context, further research is necessary to evaluate the levels of PD-1-positive IM MO levels in newborns born full term.

We observed that there was higher count of PD-1 positive NC MO subset on the 5^th^ DOL in the infants exposed to antenatal steroid administration. According to Fingerle-Rowson et al. [32], and Dayyani et al. [33], glucocorticoid treatment led to decreased number of NC MO due to a selective induction of their apoptosis. We did not observe any decline in NC MO, however our study differed from the researches cited above in the dosage and timing of steroid administration prior to analysis of MO subsets. There were conflicting studies in regard to steroid effect on the monocyte function. In animal studies by Kramer et al. [34], there was a time-dependent suppression of MO functions, such as phagocytic capacity, hydrogen peroxide production or Il-6 production, in preterm-born lambs after antenatal glucocorticoids administration. In contrast, Kavelaars et al. [35] analyzed cord blood from 38 preterm-born children whose mothers had received full course of betamethasone due to threatened preterm labor and showed lack of effect of the antenatal steroid treatment on Il-6 production by MO. Xing et al. [36], who studied immunosuppressive effects of glucocorticoids in anti-cancer therapy, showed that dexamethasone and hydrocortisone could enhance PD-1 expression both in mouse and human activated T cells in a dose-dependent manner. Further studies are needed to evaluate whether similar mechanisms are present in MO.

In our study, we observed a higher count of IM and NC MO subsets in the second blood sample collected within 24 hours after onset of LOS. Similar results were reported by Skrzeczyńska et al. [7] in their studies of the MO subsets changes during neonatal sepsis. Moreover, we observed a significant rise in percentage and absolute number of IM MO with PD-1 expression in a subgroup of patients who developed septic shock. Also, children who died in the course of septic shock had increased absolute number of PD-1 positive IM MO. Our findings were consistent with the report by Huang and colleagues [16] who demonstrated that PD-1 expression on the circulating MO was higher in adult patients with septic shock than in healthy volunteers. Similarly, Guignant et al [37]. in their study of 64 patients with septic shock reported increased expression of PD-1 and its ligands on the MO and CD4^+^T lymphocytes on days 1–2 and 3–5 after the onset of septic shock. Increased PD-1 and PD-1 ligands’ expression was associated with typical sepsis-related immune dysfunctions, such as decreased monocyte HLA-DR expression, decreased circulating CD4^+^ T-cell count, increased percentage of the T regulatory cells, as well as increased rates of secondary nosocomial infections and mortality. In contrast, the study by Zhang et al. [38] observed up-regulation of solely ligands for PD-1 (PD-L1), but not PD-1, on CD14^+^ MO in septic shock adult patients in comparison to healthy volunteers. The only study to evaluate PD-1 expression in neonatal sepsis was performed in PD-1 knockout murine neonates with sepsis caused by cecal slurry [39]; it showed improved survival of the septic PD-1 knockout mice in comparison with the wild-type. In general, it is assumed that increased activity of the PD1/PD-L1 system in sepsis could lead to depletion of the key cells, such as monocytes and T cells, necessary for proper response to infection, therefore impairing essential anti-microbial and regulatory activities [37]. Data from ex-vivo experiments [40], animal experiments [41], as well as from the clinical trials with oncologic patients [42] showed that both antibodies blocking the PD-1 pathway and selected hormones (ghrelin, growth hormone) [43] not only diminished expression of PD-1 related molecules, but also restored function of the MO, neutrophils, T cells, and natural killer cells, which may restore immune function and diminish immunosuppression during sepsis.

We also showed an increase of PD-1-positive CL monocytes. During sepsis, there is an increase in PD-1 expression on the classical MO, and, as they mature, they differentiate into CD16^+^ (intermediate and non-classical) MO. These MO subsets perpetuate shock mechanisms that develop during sepsis progression. This may be viewed as a physiological “safety mechanism”, such as, less CL MO are able to mature into pro-inflammatory IM and NC MO since they are susceptible to apoptosis at the classical stage.

In summary, the data presented herein demonstrate that 1) MO from VLBW children show expression of PD-1, 2) children whose mothers had received antenatal steroids presented with higher absolute number of PD-1 positive NC MO subset on the 5^th^ DOL, 3) extremely premature infants (born ≤27 gestational week) diagnosed with LOS during their hospitalization showed lower percentage of PD-1 positive IM MO compared to the population born after 28^th^ week of pregnancy 4) there was a rise in the percentage of PD-1 positive CL MO during LOS, 5) prematurely born infants with septic shock or fatal outcome showed a higher percentage and absolute numbers of IM MO with PD-1 expression.

We believe that further research is needed to evaluate changes in expression of PD-1 system on MO subsets during sepsis and septic shock in infant population. Identification of the factors, which may regulate and/or temporarily inhibit PD-1 receptors might provide a valuable tool to fight severe inflammatory diseases, such as neonatal sepsis.

## References

[1] Stoll BJ, Hansen NI, Sánchez PJ, Faix RG, Poindexter BB, Van Meurs KP, et al Early onset neonatal sepsis: the burden of group B Streptococcal and E. coli disease continues. Pediatrics. 2011 5;127(5):817–26 doi: 10.1542/peds.2010-2217 21518717 10.1542/peds.2010-2217 PMC3081183

[2] Levy O. Innate immunity of the newborn: basic mechanisms and clinical correlates. Nat Rev Immunol. 2007 5;7(5):379–90 doi: 10.1038/nri2075 17457344 10.1038/nri2075

[3] Ziegler-Heitbrock L, Ancuta P, Crowe S, Dalod M, Grau V, Hart DN, et al Nomenclature of monocytes and dendritic cells in blood. Blood. 2010 10 21;116(16):e74–80 doi: 10.1182/blood-2010-02-258558 20628149 10.1182/blood-2010-02-258558

[4] Ziegler-Heitbrock L. Monocyte subsets in man and other species. Cell Immunol. 2014 May-Jun;289(1–2):135–9 doi: 10.1016/j.cellimm.2014.03.019 24791698 10.1016/j.cellimm.2014.03.019

[5] Ziegler-Heitbrock L. The CD14+ CD16+ blood monocytes: their role in infection and inflammation. J Leukoc Biol. 2007 3;81(3):584–92 doi: 10.1189/jlb.0806510 17135573 10.1189/jlb.0806510

[6] Fingerle G, Pforte A, Passlick B, Blumenstein M, Ströbel M, Ziegler-Heitbrock HW. The novel subset of CD14+/CD16+ blood monocytes is expanded in sepsis patients. Blood. 1993 11 15;82(10):3170–6 7693040

[7] Skrzeczyńska J, Kobylarz K, Hartwich Z, Zembala M, Pryjma J. CD14+CD16+ monocytes in the course of sepsis in neonates and small children: monitoring and functional studies. Scand J Immunol. 2002 6;55(6):629–38 12028567 10.1046/j.1365-3083.2002.01092.x

[8] Hotchkiss RS, Coopersmith CM, McDunn JE, Ferguson TA. The sepsis seesaw: tilting toward immunosuppression. Nat Med. 2009 5;15(5):496–7 doi: 10.1038/nm0509-496 19424209 10.1038/nm0509-496 PMC3786779

[9] Pickkers P, Kox M. Towards precision medicine for sepsis patients. Crit Care. 2017 1 12;21(1):11 doi: 10.1186/s13054-016-1583-z 28077168 10.1186/s13054-016-1583-z PMC5228110

[10] Ishida Y, Agata Y, Shibahara K, Honjo T. Induced expression of PD-1, a novel member of the immunoglobulin gene superfamily, upon programmed cell death. EMBO J. 1992 11;11(11):3887–95 1396582 10.1002/j.1460-2075.1992.tb05481.x PMC556898

[11] Liang SC, Latchman YE, Buhlmann JE, Tomczak MF, Horwitz BH, Freeman GJ, et al Regulation of PD-1, PD-L1, and PD-L2 expression during normal and autoimmune responses. Eur J Immunol. 2003 10;33(10):2706–16 doi: 10.1002/eji.200324228 14515254 10.1002/eji.200324228

[12] Okazaki T, Honjo T. PD-1 and PD-1 ligands: from discovery to clinical application. Int Immunol. 2007 7;19(7):813–24 doi: 10.1093/intimm/dxm057 17606980 10.1093/intimm/dxm057

[13] Keir ME, Butte MJ, Freeman GJ, Sharpe AH. PD-1 and its ligands in tolerance and immunity. Annu Rev Immunol. 2008;26:677–704 doi: 10.1146/annurev.immunol.26.021607.090331 18173375 10.1146/annurev.immunol.26.021607.090331 PMC10637733

[14] Yamazaki T, Akiba H, Iwai H, Matsuda H, Aoki M, Tanno Y, et al Expression of programmed death 1 ligands by murine T cells and APC. J Immunol. 2002 11 15;169(10):5538–45 12421930 10.4049/jimmunol.169.10.5538

[15] Riley JL. PD-1 signaling in primary T cells. Immunol Rev. 2009 5;229(1):114–25 doi: 10.1111/j.1600-065X.2009.00767.x 19426218 10.1111/j.1600-065X.2009.00767.x PMC3424066

[16] Huang X, Venet F, Wang YL, Lepape A, Yuan Z, Chen Y, et al PD-1 expression by macrophages plays a pathologic role in altering microbial clearance and the innate inflammatory response to sepsis. Proc Natl Acad Sci U S A. 2009 4 14;106(15):6303–8 doi: 10.1073/pnas.0809422106 19332785 10.1073/pnas.0809422106 PMC2669369

[17] Brahmamdam P, Inoue S, Unsinger J, Chang KC, McDunn JE, Hotchkiss RS. Delayed administration of anti-PD-1 antibody reverses immune dysfunction and improves survival during sepsis. J Leukoc Biol. 2010 8;88(2):233–40 doi: 10.1189/jlb.0110037 20483923 10.1189/jlb.0110037 PMC6607999

[18] Zhang Y, Zhou Y, Lou J, Li J, Bo L, Zhu K, et al PD-L1 blockade improves survival in experimental sepsis by inhibiting lymphocyte apoptosis and reversing monocyte dysfunction. Crit Care. 2010;14(6):R220 doi: 10.1186/cc9354 21118528 10.1186/cc9354 PMC3220038

[19] Dellinger RP, Levy MM, Rhodes A, Annane D, Gerlach H, Opal SM, et al Surviving sepsis campaign: international guidelines for management of severe sepsis and septic shock: 2012. Crit Care Med. 2013 2;41(2):580–637 doi: 10.1097/CCM.0b013e31827e83af 23353941 10.1097/CCM.0b013e31827e83af

[20] Goldstein B, Giroir B, Randolph A; International Consensus Conference on Pediatric Sepsis. International pediatric sepsis consensus conference: definitions for sepsis and organ dysfunction in pediatrics. Pediatr Crit Care Med. 2005 1;6(1):2–8 doi: 10.1097/01.PCC.0000149131.72248.E6 15636651 10.1097/01.PCC.0000149131.72248.E6

[21] Siedlar M, Strach M, Bukowska-Strakova K, Lenart M, Szaflarska A, Węglarczyk K, et al Preparations of intravenous immunoglobulins diminish the number and proinflammatory response of CD14+CD16++ monocytes in common variable immunodeficiency (CVID) patients. Clin Immunol. 2011 5;139(2):122–32 doi: 10.1016/j.clim.2011.01.002 21300572 10.1016/j.clim.2011.01.002

[22] Heimbeck I, Hofer TP, Eder C, Wright AK, Frankenberger M, Marei A, et al Standardized single-platform assay for human monocyte subpopulations: Lower CD14+CD16++ monocytes in females. Cytometry A. 2010 9;77(9):823–30 20662093 10.1002/cyto.a.20942

[23] Yang J, Zhang L, Yu C, Yang XF, Wang H. Monocyte and macrophage differentiation: circulation inflammatory monocyte as biomarker for inflammatory diseases. Biomark Res. 2014 1 7;2(1):1 doi: 10.1186/2050-7771-2-1 24398220 10.1186/2050-7771-2-1 PMC3892095

[24] Wisgrill L, Groschopf A, Herndl E, Sadeghi K, Spittler A, Berger A, et al Reduced TNF-α response in preterm neonates is associated with impaired nonclassic monocyte function. J Leukoc Biol. 2016 9;100(3):607–12 doi: 10.1189/jlb.4A0116-001RR 26965638 10.1189/jlb.4A0116-001RR

[25] Monneret G, Gossez M, Venet F. Sepsis in PD-1 light. Crit Care. 2016 7 5;20(1):186 doi: 10.1186/s13054-016-1370-x 27378029 10.1186/s13054-016-1370-x PMC4932709

[26] Fuertes Marraco SA, Neubert NJ, Verdeil G, Speiser DE. Inhibitory Receptors Beyond T Cell Exhaustion. Front Immunol. 2015 6 26;6:310 doi: 10.3389/fimmu.2015.00310 26167163 10.3389/fimmu.2015.00310 PMC4481276

[27] Ziegler-Heitbrock L, Hofer TP. Toward a refined definition of monocyte subsets. Front Immunol. 2013 2 4;4:23 doi: 10.3389/fimmu.2013.00023 23382732 10.3389/fimmu.2013.00023 PMC3562996

[28] Zawada AM, Rogacev KS, Rotter B, Winter P, Marell RR, Fliser D, et al SuperSAGE evidence for CD14++CD16+ monocytes as a third monocyte subset. Blood. 2011 9 22;118(12):e50–61 doi: 10.1182/blood-2011-01-326827 21803849 10.1182/blood-2011-01-326827

[29] Zhao C, Tan YC, Wong WC, Sem X, Zhang H, Han H, et al The CD14(+/low)CD16(+) monocyte subset is more susceptible to spontaneous and oxidant-induced apoptosis than the CD14(+)CD16(-) subset. Cell Death Dis. 2010 11 4;1:e95 doi: 10.1038/cddis.2010.69 21368871 10.1038/cddis.2010.69 PMC3032320

[30] Wong KL, Tai JJ, Wong WC, Han H, Sem X, Yeap WH, et al Gene expression profiling reveals the defining features of the classical, intermediate, and nonclassical human monocyte subsets. Blood. 2011 8 4;118(5):e16–31. doi: 10.1182/blood-2010-12-326355 21653326 10.1182/blood-2010-12-326355

[31] Roy S, Gupta P, Palit S, Basu M, Ukil A, Das PK. The role of PD-1 in regulation of macrophage apoptosis and its subversion by Leishmania donovani. Clin Transl Immunology. 2017 5 5;6(5):e137 doi: 10.1038/cti.2017.12 28690843 10.1038/cti.2017.12 PMC5493582

[32] Fingerle-Rowson G, Angstwurm M, Andreesen R, Ziegler-Heitbrock HW. Selective depletion of CD14+ CD16+ monocytes by glucocorticoid therapy. Clin Exp Immunol. 1998 6;112(3):501–6 9649222 10.1046/j.1365-2249.1998.00617.x PMC1904988

[33] Dayyani F, Belge KU, Frankenberger M, Mack M, Berki T, Ziegler-Heitbrock L. Mechanism of glucocorticoid-induced depletion of human CD14+CD16+ monocytes. J Leukoc Biol. 2003 7;74(1):33–9 12832440 10.1189/jlb.1202612

[34] Kramer BW, Ikegami M, Moss TJ, Nitsos I, Newnham JP, Jobe AH. Antenatal betamethasone changes cord blood monocyte responses to endotoxin in preterm lambs. Pediatr Res. 2004 5;55(5):764–8 doi: 10.1203/01.PDR.0000120678.72485.19 14973182 10.1203/01.PDR.0000120678.72485.19

[35] Kavelaars A, van der Pompe G, Bakker JM, van Hasselt PM, Cats B, Visser GH, et al Altered immune function in human newborns after prenatal administration of betamethasone: enhanced natural killer cell activity and decreased T cell proliferation in cord blood. Pediatr Res. 1999 3;45(3):306–12 doi: 10.1203/00006450-199903000-00003 10088646 10.1203/00006450-199903000-00003

[36] Xing K, Gu B, Zhang P, Wu X. Dexamethasone enhances programmed cell death 1 (PD-1) expression during T cell activation: an insight into the optimum application of glucocorticoids in anti-cancer therapy. BMC Immunol. 2015 6 26;16:39 doi: 10.1186/s12865-015-0103-2 26112261 10.1186/s12865-015-0103-2 PMC4480888

[37] Guignant C, Lepape A, Huang X, Kherouf H, Denis L, Poitevin F, et al Programmed death-1 levels correlate with increased mortality, nosocomial infection and immune dysfunctions in septic shock patients. Crit Care. 2011;15(2):R99 doi: 10.1186/cc10112 21418617 10.1186/cc10112 PMC3219369

[38] Zhang Y, Li J, Lou J, Zhou Y, Bo L, Zhu J, et al Upregulation of programmed death-1 on T cells and programmed death ligand-1 on monocytes in septic shock patients. Crit Care. 2011;15(1):R70 doi: 10.1186/cc10059 21349174 10.1186/cc10059 PMC3222003

[39] Young WA, Fallon EA, Heffernan DS, Efron PA, Cioffi WG, Ayala A. Improved survival after induction of sepsis by cecal slurry in PD-1 knockout murine neonates. Surgery. 2017 5;161(5):1387–1393 doi: 10.1016/j.surg.2016.11.008 28012568 10.1016/j.surg.2016.11.008 PMC5404993

[40] Patera AC, Drewry AM, Chang K, Beiter ER, Osborne D, Hotchkiss RS. Frontline Science: Defects in immune function in patients with sepsis are associated with PD-1 or PD-L1 expression and can be restored by antibodies targeting PD-1 or PD-L1. J Leukoc Biol. 2016 12;100(6):1239–1254 doi: 10.1189/jlb.4HI0616-255R 27671246 10.1189/jlb.4HI0616-255R PMC5110001

[41] Shindo Y, McDonough JS, Chang KC, Ramachandra M, Sasikumar PG, Hotchkiss RS. Anti-PD-L1 peptide improves survival in sepsis. J Surg Res. 2017 2;208:33–39 doi: 10.1016/j.jss.2016.08.099 27993215 10.1016/j.jss.2016.08.099 PMC5535083

[42] Topalian SL, Drake CG, Pardoll DM. Immune checkpoint blockade: a common denominator approach to cancer therapy. Cancer Cell. 2015 4 13;27(4):450–61 doi: 10.1016/j.ccell.2015.03.001 25858804 10.1016/j.ccell.2015.03.001 PMC4400238

[43] Zhou M, Yang WL, Aziz M, Ma G, Wang P. Therapeutic effect of human ghrelin and growth hormone: Attenuation of immunosuppression in septic aged rats. Biochim Biophys Acta. 2017 1 20 pii: S0925-4439(17)30027-3. [Epub ahead of print] doi: 10.1016/j.bbadis.2017.01.014 28115288 10.1016/j.bbadis.2017.01.014 PMC5519455

